# Pathophysiology and Therapeutic Management of Bone Loss in Patients with Critical Illness

**DOI:** 10.3390/ph16121718

**Published:** 2023-12-11

**Authors:** Taejin Kim, Hyojin Kim

**Affiliations:** 1 Department of Urology, CHA University Ilsan Medical Center, CHA University School of Medicine, Goyang-si 10414, Republic of Korea; tjkim81@cha.ac.kr; 2 Division of Critical Care Medicine, Department of Anesthesiology and Pain Medicine, Chung-Ang University Gwangmyeong Hospital, Gwangmyeong-si 14353, Republic of Korea

**Keywords:** bone loss, critical illness, fracture, osteoporosis

## Abstract

Patients with critical illnesses are at higher risk of comorbidities, which can include bone mineral density loss, bone turnover marker increase, and fragility fractures. Patients admitted to intensive care units (ICUs) have a higher risk of bone fractures. Since hypermetabolism is a characteristic of ICU patients, such patients are often rapidly affected by systemic deterioration, which often results in systemic wasting disease. Major risk factors for ICU-related bone loss include physical restraint, inflammation, neuroendocrine stress, malnutrition, and medications. A medical history of critical illness should be acknowledged as a risk factor for impaired bone metabolism. Bone loss associated with ICU admission should be recognized as a key component of post-intensive care syndrome, and further research that focuses on treatment protocols and prevention strategies is required. Studies aimed at maintaining gut integrity have emphasized protein administration and nutrition, while research is ongoing to evaluate the therapeutic benefits of anti-resorptive agents and physical therapy. This review examines both current and innovative clinical strategies that are used for identifying risk factors of bone loss. It provides an overview of perioperative outcomes and discusses the emerging novel treatment modalities. Furthermore, the review presents future directions in the treatment of ICU-related bone loss.

## 1. Introduction

Critically ill patients encounter physical symptoms and physiological changes of varying severity and persistence [1]. Compared with the general population and regardless of their premorbid health status, patients with critical illness experience increased mortality [1,2,3], physical impairment [4,5], cognitive impairment [6,7], and psychological distress [8,9]. Critically ill patients experience physical manifestations, such as muscle loss or atrophy, as well as decreased autonomy in carrying out activities of daily living. An increased incidence of fragility fractures is one area in which critical illness may negatively impact patients’ well-being [10,11]. Moreover, clinical studies have indicated that bone health and metabolism are affected in critically ill patients, among whom the pathophysiologic mechanisms are often distinct from the mechanisms underlying the original etiology for intensive care unit (ICU) admission. Although clinical research on bone alterations during critical illness is ongoing, data are limited on this disease entity, and its prevention and treatment are limited in the field of critical care medicine. This review provides an overview of the alterations in bone metabolism in patients undergoing critical care, summarizes clinical outcomes for current therapies for bone loss in critically ill patients, and examines emerging novel therapeutic options for improving outcomes associated with critical bone loss.

## 2. Bone Metabolism and Remodeling in the Disease Setting of Critical Illness

There is a growing body of clinical data and research suggesting associations between critical illness and altered bone metabolism, ultimately resulting in bone loss and osteoporosis. The main risk factors for osteopenia are aging, female sex, low body weight or body mass index, mobility limitations, smoking, and chronic illness. Clinical factors that contribute to bone loss in ICU patients include inflammation, neuroendocrine factors, physical immobilization, malnutrition (including vitamin D deficiency), and medications (including corticosteroids and diuretics) [12]. Various studies have demonstrated associations between increased bone turnover rates and critical illness. An increase in bone resorption characterized by elevated levels of bone turnover markers (BTM) occurs during the first 2 to 3 weeks after a critical event, while a decrease in bone mineral density (BMD) and an increased risk of fragility fractures persists for up to 2 years after admission to an ICU [13,14,15].

By inhibiting osteoclastic activity while enhancing bone formation, hormonal factors that include estrogen, testosterone, and parathyroid hormone (PTH) contribute significantly to the process of bone remodeling. Women reach peak bone mass at the age of 25–30 years [16]. The decrease in estrogen synthesis in postmenopausal women is a major cause of bone loss and reduced bone metabolism. Although increased rates of bone loss are observed during perimenopause, the rate of bone loss decreases several years after menopause [17]. Decreased bone remodeling in the menopausal population affects both cancellous and cortical bone, along with the weakening of bone microarchitecture and bone loss. Trabecular thinning is observed in cancellous bone, whereas reduced cortical thickness and an increase in cortical porosity are predominant characteristics of the cortical bony skeleton [17,18]. In comparison, a gradual decrease in BMD is observed in the male population. With aging, sex-hormone-binding globulin (SHBG) inactivates testosterone, and the relative increase in serum estrogen levels results in the decline of BMD [16]. Moreover, bone loss is predominately related to reduced bone formation and low bone turnover in men. Because of age-related hormonal changes, both male and female populations have equal rates of bone loss by the age of 60 years and are at risk of developing osteoporosis [17,18].

Glucocorticoids are the most common cause of secondary or drug-induced osteoporosis. Long-term administration of glucocorticoids can result in complications such as glucocorticoid-induced osteoporosis (GIO) [19,20]. Glucocorticoid therapy enhances osteoclast differentiation and maturation of osteoclasts while inhibiting bone formation by inducing osteoblast and osteocyte apoptosis, which ultimately results in increased bone resorption and diminished bone formation [21,22]. Moreover, glucocorticoids suppress insulin-like growth factor-1 (IGF-1), which promotes bone formation by activating type I collagen synthesis, which also leads to collagen degradation and osteoblast apoptosis. In patients with GIO, a rapid decline of BMD has been observed within three to six months of beginning of glucocorticoid therapy [23].

The disease spectrum of critical illness encompasses weight loss, increased energy expenditure, a catabolic state with protein consumption, and profound hypermetabolism [24]. Hypermetabolism in the ICU population results in a systemic wasting disorder, which is characterized mainly by degradation of the skeletal muscle and bony skeleton [25]. Molecular mediators such as tumor necrosis factor-α (TNF-α), interleukin (IL)-1, and IL-6 stimulate osteoclastogenesis while inhibiting osteoblastogenesis, which provides an environment for uncoupled bone hyperresorption [26]. This increase in bone degradation instigates the release of resorptive bone markers and triggers osteoclasts to secrete cytokines with chemotactic and immune-activating properties, thereby forming a hyperresorption setting. These result in both acute and chronic deleterious consequences and could potentially increase mortality in patients with critical illness [25]. This evolving insight and further understanding impels a change in the paradigm of the impact of critical illness on bone health and suggests an interaction between hypermetabolism and altered bone metabolism.

Bone formation and remodeling are regulated by mechanical forces and biological factors, such as microfracture repair, maintenance of mineral homeostasis, and physical weight bearing. In critically ill patients, bone metabolic activity is characterized by an increase in the number of immature osteoblasts and an escalated concentration of bone resorption markers [27]. These physiological conditions lead to an uncoupling between bone formation and resorption, which ultimately results in increased bone turnover activity [28]. To establish baseline assessment and diagnosis for osteoporosis, dual-energy X-ray absorptiometry is used to measure BMD at the proximal femur and lumbar spine. BMD assessments are expressed as T-scores, and osteopenia is defined as T-scores between 1.0 and 2.5 standard deviation units below the norm, while osteoporosis is defined as a T-score ≥2.5 standard deviations below the mean value for healthy individuals [29]. Several studies have explored associations between bone loss and critical illness. A prospective study conducted by Orford et al. showed that BMD losses among critically ill patients during the year following critical care were greater than those of age- and sex-matched controls [12]. Biannual follow-up clinical studies of ICU patients have shown that the time period of BMD loss varies between the sexes. In one study, while an increased proportion of female patients exhibited acute BMD loss during the first post-ICU discharge annual follow-up, males who had previously been admitted to ICUs with critical illness exhibited advanced osteopenia or osteoporosis in the second year following ICU discharge [15]. A growing body of clinical evidence substantiates the notion of accelerated bone loss in the context of critical illness. Moreover, most ICU survivors exhibit osteopenia or osteoporosis after acute care, which implicates a disease entity that may potentially affect long-term morbidity and mortality in this select patient group. However, due to limitations and difficulties associated with conducting long-term clinical studies investigating ICU patients, risk factors that are associated with critical illness and accelerated bone loss are yet to be determined.

## 3. BTMs in Critical Illness

With recent advances in molecular science, the detection and measurement of bone metabolism is possible via testing of BTMs [27]. BTM assessments allow physicians to observe physiological changes in bone metabolism among ICU patients [30]. There are two distinct classifications of bone markers, representing osteogenesis and bone resorption, respectively. Bone formation markers are produced from the osteoblastic activity while directly or indirectly reflecting the status of bone formation. These markers include osteocalcin (OC), PICP (C-terminal propeptide of type I procollagen), PINP (procollagen type 1 N-terminal propeptide), and BSAP (bone alkaline phosphatase). Conversely, bone resorption markers denote osteoclast activity and are indicative of bone tissue that has been metabolized or secreted during bone resorption. These markers consist of pyridinoline (PYD), hydroxyproline (HYP), N-terminal telopeptide (NTX), deoxypyridinoline (DPD), C-terminal telopeptide (CTX), matrix-metalloprotease (MMP)-generated (CTX-MMP or ICTP) type I collagen fragments, along with osteoclast-secreted bone markers, such as tartrate-resistant acid phosphatase 5b isoform (TRAP-5b), as well as the receptor activator of nuclear factor NF-kB ligand (RANKL) [31].

Various physiological conditions initiate uncoupling of bone resorption and development. Changes in serum hormone levels—such as those caused by menopause, androgen deprivation therapy for prostate cancer patients, and hormone administration for breast cancer treatment—are considered risk factors. On the other hand, conditions such as myeloma, rheumatoid arthritis, bone metastases, and increased proliferation of cytokines (such as interleukin 1 or tumor necrosis factor) are related to the uncoupling of bone metabolism [32]. Low serum estrogen levels increase bone remodeling and bone resorption by reducing the osteoblast population while promoting osteoclast activity. This physiological environment induces trabecular thinning, a disruption of connectivity in the trabecular regions, cortical thinning, and increased cortical porosity. Given these hormonal shifts and the slower rate of change in serum sex hormone levels in males, which does not result in a concurrent rise in bone remodeling, female patients exhibit a higher incidence of bone fragility [33].

According to a systematic review by Orford and colleagues, various studies have indicated the prognostic value of BTM levels among critically ill patients who have undergone mechanical ventilation for longer than 24 h [10]. The review demonstrated a moderate association between increased bone turnover and critical illness. Moreover, the results from the reviewed studies described BTM changes consistent with the uncoupling of bone metabolism in the context of critical illness. These changes consisted of an increased abundance of immature osteoblasts with reduced activity of mature osteoblasts, along with increased osteoclast activity and bone resorption. The analysis further explored the correlation between increases in BTMs associated with other factors, including the duration of illness, systemic inflammation, sepsis, and the hormonal imbalance caused by atypical regulation of the hypothalamic–pituitary axis [34,35,36,37,38]. The review concluded that with the incomplete understanding of the confounding effects of other variables, such as preexisting comorbidities, organ failure, and medication, further studies are warranted to fully comprehend the aforementioned factors and their impact on BTMs.

There is an increasing amount of clinical evidence indicating a close correlation between BTM levels and critical illness. Via et al. conducted a double-blind, randomized control study of intravenous ibandronate administration for an 11-day period in 20 postmenopausal women admitted to an ICU for longer than 5 days. At study enrollment, participants had high serum levels of CTX, which is a marker of osteoclast activity, and low osteoblast activity, reflected in the low initial serum OC levels [39]. The Correction of Vitamin D Deficiency in Critically Ill Patients (VITdAL-ICU) study was a randomized placebo-controlled trial that evaluated the benefits of vitamin D_3_ in high doses and its effect on BTMs, specifically CTX and OC in ICU patients [40]. The study demonstrated that the high CTX levels and low OC levels detected in all study participants at enrollment normalized after 6 months. A prospective observational cohort study investigating ICU patients who underwent mechanical ventilation for more than 24 h demonstrated high baseline CTX levels during ICU admission. The median level of 579 ng/L was above the upper quartile of the normal range, but this returned to normal after 1 year. In comparison, the median level of the bone formation marker P1NP was in the high-normal range during admission, and the serum P1NP levels were within normal limits during ICU admission and at follow-up after 1 year [12].

Although BTMs show potential as a prognosticator for bone loss in the critically ill population, several limitations concerning the usage and understanding of these biomarkers should be addressed. Further research is needed to fully elucidate the pathophysiological interactions of BTMs in the context of critical illness. Clinical factors affecting BTM function, including nutrition, changes in body fluid composition, systemic organ failure, and medication, should be studied further and analyzed in depth [27]. Moreover, formal quantification of the relationship between serum BTM elevations and subsequent bone loss, along with mortality, has yet to be performed in the context of critical illness [10,41].

Nevertheless, the serological properties of BTMs observed in the ICU setting are consistent with the uncoupling of bone formation and resorption. Bone metabolism uncoupling indicates rapid bone loss in the early phases of critical illness. This abnormal bone metabolism continues for weeks to months and returns to normal levels over the following year. Although research is ongoing to understand bone loss during critical illness, more clinical trials are required to elucidate the severity and period of altered bone metabolism, the osteological effects of confounding factors, and the impacts of these processes on osteogenesis in critically ill patients.

## 4. The Aftermath of Altered Bone Metabolism in ICU Patients

Due to technological advances in medicine and treatment, the increased survival rate of critically ill patients following intensive care has shown promise in the modern clinical setting [42]. Due to this success, improving quality of life and reducing disease burden in ICU survivors has become a novel topic of research. Of these novel topics, the further elucidation of bone metabolism and the prevention of fragility fractures during recovery from critical illness is ongoing. The sequelae after intensive care that patients experience is known as post-intensive care syndrome (PICS). An updated definition of this syndrome, which previously included muscle weakness, mental disorders, and cognitive dysfunction, has expanded to include accelerated bone loss and fragility fractures [43]. Fracture prevention requires a multidisciplinary methodology that is composed of early mobilization, physical rehabilitation, education on fall prevention, a tailored administration of bone-specific medication, and nutritional intervention, including an adequate intake of proteins. Critical illness results in accelerated bone loss, leading to an increased risk for fragility fracture in the post-ICU setting. Although there is an accumulating amount of clinical evidence linking critical illness and bone loss, further studies and future trials are warranted to further our understanding of underlying causative factors and treatment efficacy of preventive measures.

### 4.1. Fragility Fractures in Critically Ill Patients

An increased risk of fragility fractures due to accelerated bone loss is a major adverse event commonly affecting critically ill patients. Several studies have described an increased risk of fragility fractures in ICU survivors, and this association has been shown to be significantly higher among older women [11,44]. Orford et al. conducted a retrospective longitudinal case study that compared the incident fracture rate of female ICU survivors who were ventilated for more than 24 h with that of the women included in the Geelong Osteoporosis Study. During median follow-up periods of 3.7 years (interquartile range, 2.0–5.9 years) for women and 4.0 years (interquartile range, 2.1–6.1 years) for men in the ICU survivor cohort, 36 women (14.2%) were diagnosed with bone fractures during the post-intensive care period. The incident fracture rates were 3.84 and 2.41 per 100 patient-years for men and women, respectively. Among older female ICU survivors, there was a surge in the osteoporosis-related fracture rate and a decreased time to fracture was observed relative to that of the control cohort (hazard ratio 1.65; 95% confidence interval (CI) 1.08–2.52; *p* = 0.02) [11].

A Belgian retrospective study assessed the correlation between bone fractures and prolonged ICU admission. The study cohort consisted of patients admitted to the ICU for longer than 7 days and those who were alive after 2 years of follow-up. The 178 survivors after 2 years were compared with the control group, which comprised age- and gender-matched patients who underwent surgery [45]. At the 2-year follow-up mark, the clinical fracture rate was 5% in the study group compared with 3.4% in the control group, where all new fracture incidences were associated with fall injuries. Although the difference was not statistically significant, the ICU cohort had a 50% higher risk of new fracture occurrence (odds ratio (OR) 1.53; 95% CI 0.62–3.77; *p* = 0.35). Moreover, the fracture risk in the 2 years following prolonged ICU admission was not statistically significant.

Current evidence suggests that fragility fractures, along with elevated BTMs, are associated with various medical conditions, such as immobilization, limited exposure to sunlight, malnutrition, systemic inflammation, and endocrine disruption [28]. Although BTMs and imaging studies, such as dual-energy X-ray absorptiometry, show potential as prognosticators for bone loss, there is no formal and accurate method for quantifying and predicting fracture risk among ICU survivors. Nonetheless, alternate diagnostic methods, such as the Fracture Risk Assessment Tool (FRAX), can be used for high-risk ICU patients to screen for fragility fractures [45]. Moreover, fall injuries have been associated with fragility fractures, which are common among ICU patients, either during hospitalization or after discharge [46,47]. Additionally, most ICU patients are immobile. Oppl et al. observed a connection between spontaneous fractures and immobilization [48]. The prevalence of malnutrition in intensive care units has been reported to range from 38% to 78% and is independently associated with poor outcomes [49]. Critically ill patients in ICUs are often not exposed to sufficient natural sunlight throughout the day. As a result, they may experience a disruption in their circadian rhythms and sleep patterns, potentially leading to hormonal imbalances [50]. Although some clinical evidence exists regarding fragility fracture rates after intensive care, there is strong evidence indicating higher rates of fragility fractures during ICU admissions, with older women having the highest risk. Future clinical trials are warranted to enhance our understanding of fracture rates following critical illness and to shed light on the deleterious effects that such illnesses can have on bone metabolism.

### 4.2. Morbidity and Mortality with Increased Bone Loss 

Osteoporosis can be a serious health risk that exposes patients in various disease states to increased morbidity and mortality. Studies have reported clinically significant associations between cardiovascular disease and osteoporosis among both men and women. Moreover, the shared pathological pathways of osteoporosis and cardiovascular disease both involve inflammation, hindering osteoclastic activity and disrupting the maturation of osteoclasts, which ultimately leads to increased morbidity and mortality [41,51,52,53]. Other studies have demonstrated the clinical relevance of elevated BTMs, including in terms of mortality. Two studies by Lerchbaum et al. investigated associations between elevated BTMs and mortality among men and women undergoing coronary angiography [51,52]. Both studies concluded that there is an independent association of high BTM levels with all-cause and cardiovascular mortality. Osteocalcin has been identified as a protein that not only serves as a marker for bone formation but also plays a crucial role in regulating endothelial homeostasis, making it a potential marker for vascular calcification [54,55]. Concurrently, B-CTX, recognized as a bone resorption marker and a biomarker for collagen turnover, has been associated with cardiovascular death and heart failure in patients with acute coronary syndrome [56]. However, the precise mechanisms through which BTMs contribute to cardiovascular risk remain unclear. Nevertheless, an intriguing observation of a negative correlation between coronary artery calcium score and BTMs suggests a potential association with the formation of plaques in coronary arteries [57]. Meanwhile, the Zoledronic Acid Bone Markers (ZOMAR) study was a Spanish multicenter trial that demonstrated a significant association between increased BTM levels and mortality among patients with metastatic breast cancer [41].

Fragility fractures are common among critically ill patients following admission or after recovery and discharge [46]. In particular, there is an established link between fragility fractures caused by osteoporosis and increased mortality. A recent Canadian study showed that osteoporosis-associated fragility fractures increased the mortality rate among patients aged 50 years and older [58]. When mortality rates were analyzed according to specific fragility fracture sites, there was a 2- to 4-fold increase in mortality risk after hip, humerus, or vertebral fractures, while fractures of other sites—such as the wrists or pelvis—were associated with a 1.4- to 2-fold increase in mortality risk [58,59]. Additionally, Bliuc et al. observed an increased mortality risk lasting for 5 years after the index fracture for all fragility fractures, along with a 10-year mortality risk increase for patients with hip fractures. This tendency was accentuated for women, among whom the increases in absolute mortality ranged from 1.32 to 13.2 per 100 person-years, which was much higher than anticipated [60].

Patients in the critically ill setting are more susceptible to an increased risk of fragility fractures. Moreover, evidence increasingly suggests that bone loss in the ICU is consistent with accelerated bone turnover, and this may be associated with mortality in this specific patient population. However, further research is warranted to further explore the connection between fragility fractures and mortality rates in ICU survivors.

## 5. Lifestyle Modifications and Prevention Strategies

The requirement for comprehensive, interdisciplinary, and multimodal prevention strategies is underscored by the consequences of bone loss following critical illness. These consequences include healthcare costs, as well as factors that affect quality of life and the complex interactions between bone and muscle. It is imperative that such strategies be initiated at the early stages of critical illness [44]. With an increasing number of ICU survivors, long-term management strategies for bone loss among critically ill patients are also required. Physicians should be guided by the literature and clinical trial data, including the guidance summarized in Box 1, in their understanding and management of bone loss and osteoporosis, both generally and in the ICU context.

Box 1 **Strategies for managing bone loss in critically ill patients.**

1. Consideration of bone loss in critically ill patients should be integrated into the framework of post-ICU syndrome.
2. We strongly recommend conducting well-structured, sufficiently powered, and meticulously executed randomized controlled trials. These trials should compare early rehabilitation or proactive pharmacological interventions with standard care for critically ill patients, with a focus on measuring and reporting outcomes of significant clinical relevance to patients.
3. We suggest identifying and evaluating risk factors for ICU-associated bone loss, including:
   - Duration of ICU stay;
   - Severity of illness;
   - Immobilization;
   - Medications causing bone loss (e.g., glucocorticoids);
   - Malnutrition;
   - Endocrine dysfunction;
   - Postmenopausal status.
4. We suggest conducting radiological and serological diagnostic tests to assess bone mass status as early as possible, considering patients’ clinical conditions during their ICU stays.
5. Strategies for preventing and treating bone loss in critically ill patients emphasize a comprehensive, multidisciplinary approach.
6. Early mobilization and rehabilitation are recommended.
7. Early assessment of nutritional status upon admission to the ICU is advisable, along with the provision of appropriate nutritional support (protein, vitamin D, calcium, and micronutrients).
8. Consider pharmacological interventions for high-risk patients.

### 5.1. Early Mobilization and Rehabilitation

Despite clinical evidence indicating that prolonged bed rest and immobilization are associated with deleterious outcomes and complications, such as ICU-acquired weakness (ICUAW) and osteoporosis [61], there have been many obstacles to implementing early mobilization and rehabilitation protocols for critically ill patients [62]. Bailey et al. were the first to report on the viability and safety of early activity among ICU patients with respiratory failure [63]. Their prospective cohort study included 103 respiratory ICU patients requiring mechanical ventilation for more than 4 days. The activity protocol comprised three events: bed sitting, chair sitting, and ambulation. After 1449 activity events were completed by 103 patients, the activity results consisted of 233 instances of bed sitting (16%), 454 instances of chair sitting (31%), and 762 instances of ambulation (53%). Adverse events related to each activity were documented according to six specific indicators: fall to the knees, removal of a feeding tube, systolic blood pressure exceeding 200 mmHg, systolic blood pressure falling below 90 mmHg, oxygen desaturation below 80%, and unexpected extubation. The rate of adverse events during the study period was less than 1%. The study concluded that early activity has potential benefits in the ICU population. Additionally, various clinical studies involving early mobilization and rehabilitation during the initial stages of critical illness have indicated that this therapeutic approach is both safe and well tolerated among ICU patients [64,65,66,67,68]. Several meta-analyses have indicated that the early mobilization and rehabilitation of critically ill patients reduces the incidence of ICUAW and improves short-term survival rates [69,70,71,72,73]. To our knowledge, no published studies have investigated the association between early rehabilitation interventions and bone density among critically ill patients. Nonetheless, the 2018 ICU guidelines released by the Society of Critical Care Medicine place a significant emphasis on early mobilization and rehabilitation [74]. These recommendations are grounded in the goals of enhancing muscle strength at the time of ICU discharge, reducing the duration of mechanical ventilation, and enhancing health-related quality of life. Although early mobilization and physical therapy in the ICU setting have therapeutic benefits on the outcomes of critically ill patients, there are limitations and barriers to consider and overcome. ICU-related processes and medical structures are common limitations in the clinical setting, and through examination of these factors, should be addressed for the implementation of early rehabilitation. Individualized treatment and tailoring therapeutic modalities require several factors to be considered. Issues including specific patients, which treatment modalities, and which co-interventions are most likely to be of therapeutic benefit need further elucidation. A recent explorative trial showed that muscle-activating measures may augment the positive effects of physiotherapy on myocytes and have the potential to mitigate muscle wasting [75]. Although previous studies have shown the positive metabolic effects of electrical muscle stimulation (EMS) in muscle cells [76,77], underlying comorbidities and the severity of the illness influence the contractile response to EMS and treatment benefits [78]. 

### 5.2. Cessation of Alcohol Intake and Cigarette Smoking

The intake of alcohol has shown dose-dependent physiological effects in regard to bone metabolism. When consumed in light or moderate doses, alcohol functions as a preventive measure of BMD, while in comparison, high dosages of alcohol consumption are considered a risk factor for fracture [79]. Study results show that the positive effect of alcohol on bone health could be due to its acute suppressive nature on bone resorption, as evidenced by the low levels of CTX associated with ethanol intake [80,81,82], while a high dosage of alcohol disrupts serum calcium homeostasis by interfering with calcium absorption in the intestinal system, along with reducing vitamin D production and increasing the risk of injury and falls [80].

A negative and independent relationship between cigarette smoking and bone health has been evidenced in various clinical studies [83]. In a study performed by Kanis et al., results showed that cigarette smoking is associated with loss of bone mass and the risk of fracture in the elderly population [84]. Moreover, this association has been observed in female smokers with the consequential effects on bone health [85]. The negative influence of cigarettes on bone loss can be explained by the free radicals that are generated with smoking, which leads to an increase in the production of enzymes that destroy estrogen, which results in hindering the bone remodeling cascade [86]. In addition, smoking induces inflammatory reactions and increases oxidative stress, which causes damage to collagen synthesis and increases resistance to calcitonin. These molecular alterations impede bone angiogenesis, which would alter the flow of oxygen and nutrients to the bone [87].

### 5.3. Nutritional Support

The importance of nutritional support is an evolving issue in the field of critical care medicine. Optimization and personalization of nutritional support both during and after ICU admission play pivotal roles in reducing the risks of malnutrition, muscle wasting, and bone loss among critically ill patients, who are typically severely catabolic [88]. A large retrospective observational study conducted in the United States, including 6518 patients, evaluated the association between nutritional status and mortality [89]. In the cohort, 56% exhibited nonspecific malnutrition, 12% had protein-energy malnutrition, and only 32% were adequately nourished. Nutritional status was shown to be a significant predictor of 30-day mortality among critically ill patients. The OR for 30-day mortality among patients with protein-energy malnutrition was twice as high as that among patients without malnutrition (OR 2.10; 95% CI 1.70–2.59). During critical illness, rapid proteolysis and muscle wasting occur, and these are key contributors to the development of ICUAW [90]. A high-dose protein intake of 1.3 g/kg per day has been strongly recommended to prevent metabolic adverse events in the context of critical illness [24]. A meta-analysis revealed a positive correlation between high-dose protein intake and BMD [91]. Essential fatty acids in phospholipids play a role in promoting regular growth [92], and in animals, they are also linked to maintaining normal bone mass [93]. Studies using animal models suggest that omega-3 polyunsaturated fatty acids (Ω-3 PUFAs) have the potential to inhibit osteoclast activity and enhance osteoblastic activity [94,95,96]. Lately, there have been suggestions to enhance bone health and lower the risk of osteoporosis through nutritional supplements, including Ω-3 PUFAs, along with calcium and vitamin D [97,98,99]. A meta-analysis of 12 RCTs on the effects of Ω-3 PUFAs on bone health showed that n-3 PUFAs can slightly improve bone mineral density (BMD) levels (0.005 g/cm^2^; 95% CI, 0.000–0.010) [100]. To date, studies concerning Ω-3 PUFAs and bone health in critically ill patients are limited. Further clinical studies are required to clarify the relationship between nutritional provision, including a high-protein diet, and bone health in critically ill patients.

Although there is a limited amount of direct clinical data relating bone loss to the status of the gut microbiome, indirect evidence indicates that the microbiome influences bone mass and quality [101]. Experimental data have shown that modulation of gut microbiota with probiotics administration can increase bone mass [102]. Pharmacological therapy using prebiotics leads to enhanced calcium absorption and favorable changes in gut microbiota composition, resulting in improved bone mass [103,104]. Moreover, numerous endogenous and iatrogenic factors, such as gastrointestinal disorders, administration of antibiotics, proton pump inhibitors, and opioids, along with enteral feeding, can further influence the progressive disruption of the gut microbiota in the ICU population [104]. Therefore, further research is required to understand the effects of gut microbiology on bone health, and future clinical trials on microbiome modulation are needed to develop novel adjuvant therapeutic modalities for the prevention and treatment of ICU-related osteoporosis or osteoporosis.

## 6. Pharmacological Intervention and Treatment of Osteoporosis 

### 6.1. Vitamin D

High prevalence—ranging from 40% to 70%—of vitamin D deficiency has been noted among critically ill patients [105]. Traditionally, vitamin D has been considered an essential component of osteoporosis prevention and treatment. However, a meta-analysis of the effects of vitamin D supplementation on BMD conducted by Reid and colleagues revealed surprising results [106]. In their systematic review, which included 23 randomized controlled trials (RCTs) and more than 4000 participants, the investigators reported that in a biannual follow-up after vitamin D supplementation, no differences in BMD in the major bone regions (lumbar spine, entire hip joint, forearm, and total body) were observed, with the exception of a significant increase observed in the femur neck (mean weight difference 0.8%, 95% CI 0.2–1.4). The authors established that there is no compelling reason to recommend the widespread administration of prophylactic vitamin D for healthy adults [107]. The effects of vitamin D administration on serum BTM levels in critically ill patients have shown disparities in different studies, suggesting that accelerated bone turnover in critical illness is a complex and multifactorial phenomenon [35,40,108]. The Early High-Dose Vitamin D_3_ for Critically Ill, Vitamin D-Deficient Patients (VIOLET) trial (NCT03096314), a randomized placebo-controlled clinical trial investigating an early high-dose vitamin D_3_ (single enteral dose of 540,000 IU) intervention for critical illness, was prematurely terminated after it enrolled one-third of the planned patient sample of 3000 [109]. In the VIOLET trial, the early administration of high-dose enteral vitamin D_3_ did not yield any significant advantages over a placebo concerning various outcomes, including 90-day mortality, among critically ill patients with vitamin D deficiency. This also pertained to nonfatal outcomes, such as the incidence of fall-related fractures.

### 6.2. Alteration of The Hypermetabolic State of ICU Patients 

Gamrin and colleagues showed that the administration of recombinant human growth hormone (rhGH) has induced muscle protein synthesis and has protein-sparing effects in the clinical setting of critical illness [110]. Due to the lack of clinical benefits, such as improving muscle function and the risk of increased mortality, rhGH is not considered a first-line treatment of choice in ICU patients [111,112]. However, rhGH administration after discharge increased lean body mass, bone mineral content, and height without any adverse outcome in pediatric patients with thermal injuries [113]. Moreover, oxandrolone, which is a synthetic testosterone analog, showed promising treatment properties. A recent meta-analysis performed by Ring et al. showed that it does not affect mortality while increasing lean body mass and BMD in patients suffering from severe burns [114]. Given that rhGH is not currently suitable for the treatment of bone loss in ICU survivors, indirect clinical evidence shows that the blunting of adrenergic activity may yield positive outcomes in protein metabolism and increase BMD in critically ill patients with hypermetabolism.

### 6.3. Anti-Resorptive Agents

#### 6.3.1. Bisphosphonates

Bisphosphonates are widely used as first-line agents to prevent fractures and treat osteoporosis [115]. Bisphosphonates induce apoptosis in osteoclasts, which are involved in the degradation of minerals on the bone surface [116]. A retrospective study of 50 chronically and critically ill patients who were dependent on ventilator support revealed that the concurrent administration of oral pamidronate and calcitriol led to a noteworthy reduction in urinary NTX, a biomarker of bone resorption [35]. Via and colleagues conducted a prospective, double-blind, placebo-controlled therapeutic trial investigating the effect of single-dose intravenous ibandronate (3 mg) on bone resorption in 20 postmenopausal women with chronic critical illness [39]. The group treated with ibandronate exhibited a 34% reduction in serum CTX levels, indicative of reduced osteoclast activity, compared with a 13% increase in the placebo group (*p* = 0.01). However, a limitation of the treatment is that this effect is temporary. An Australian retrospective study found that the administration of bisphosphonate was associated with a survival benefit (reduction in mortality ratio 0.38, 95% CI 0.20–0.71, *p* < 0.01) among critically ill patients [117]. Furthermore, there was significant attenuation of the extent of vertebral BMD decline during ICU admission among patients taking bisphosphonate relative to non-users, with values of −3 ± 13% vs. −15 ± 14% per week, *p* < 0.01). Orford et al., in their prospective study including 92 critically ill patients, observed an association between anti-fracture therapy and changes in BMD [15]. Over a 2-year study period, 10 of 92 participants were prescribed anti-fracture medication, of which alendronate (a second-generation bisphosphonate) was prescribed to 5 of 10 patients. The study observed an increase in BMD among patients receiving anti-fracture therapy, in contrast to a decrease in BMD among those not receiving anti-fracture medication (femur 3.1 ± 2.4% vs. −2.8 ± 1.7%, *p* = 0.04; spine 5.1 ± 2.5% vs. −3.2 ± 1.8%, *p* = 0.01). Overall, the administration of bisphosphonates offers the advantage of reducing or attenuating bone loss and potentially improving survival outcomes. However, the available clinical evidence supporting these therapeutic benefits is limited.

Long-term administration of bisphosphonates is generally well tolerated by the patient and is considered safe and effective [118]. However, the most common adverse event of bisphosphonate treatment is gastrointestinal irritation. Adverse reactions in the upper gastrointestinal tract may include dysphagia, achalasia, esophageal erosion, or esophageal ulcers [119]. Furthermore, bisphosphonate therapy has been associated with occasional severe side effects, which include osteonecrosis of the jaw (ONJ) and atypical femoral fractures.

ONJ is a serious but rare effect of anti-resorptive agents or angiogenesis inhibitors. This disease entity is characterized by progressive bone destruction in the maxillofacial area of patients without a prior history of radiation therapy or metastatic disease in the mandibular area [120]. According to a study performed by Lo et al., the prevalence of bisphosphonate-associated ONJ in patients with long-term therapy has been estimated to be 0.21% over four years of therapy [121]. A single-center study showed that dental management by the screening and treatment of oral diseases concomitantly with the treatment with ONJ-related pharmacotherapy can significantly prevent the aforementioned adverse event [122].

Atypical femoral fractures are observed mainly along the femoral diaphyseal, which is anatomically in the subtrochanteric region and occurs with little trauma. In a study aimed to evaluate the risk of atypical femur fracture in female patients undergoing bisphosphonates over a ten-year period, the risk of atypical femur fracture increased with longer durations of bisphosphonate use and rapidly decreased after the cessation of bisphosphonate therapy [123]. However, after a 10-year follow-up, the same study concluded that bisphosphonate treatment decreased the risk of osteoporotic and hip fractures, which outweighed the increased risk of atypical fractures in the female osteoporotic population [123]. 

#### 6.3.2. Denosumab

RANKL is a strong regulator of bone resorption [124]. Denosumab is a human monoclonal anti-RANKL antibody, approved for the treatment of osteoporosis in 2010 on the basis of its potent anti-bone resorptive activity [125]. The sole study investigating the relationship between denosumab administration and bone loss in critically ill patients was carried out by an Austrian research group. Wadiura and colleagues conducted a phase II clinical trial involving 14 previously healthy patients admitted to the ICU [126]. The investigators used a randomized, double-blind, placebo-controlled, non-inferiority design to assess whether prophylactic administration of denosumab is effective at preventing changes in bone turnover caused by acute immobilization. The mean temporal changes in the levels of PICP, an osteolysis biomarker, over 4 weeks of denosumab administration were −0.45 mg/mL (95% CI −0.72 to −0.18) in the denosumab group and 0.29 ng/mL (95% CI −0.01 to 0.58) in the placebo group; the intergroup difference was statistically significant. The authors concluded that prophylactic denosumab monotherapy, administered soon after ICU admission, is effective at preventing acute increases in bone resorption related to immobilization among previously healthy patients. The Bone Loss Prevention with Zoledronic Acid or Denosumab in Critically Ill Adults (BoneZone) study (NCT04608630) is a multicenter, prospective, phase II, double-blind clinical trial evaluating the effect of denosumab or zoledronic acid, compared with placebo, on BMD changes in patients requiring ICU admission for longer than 24 h [127]. With an estimated number of 450 participants, the trial will randomly assign patients to denosumab, zoledronic acid, and placebo arms. The patients will receive denosumab, zoledronic acid, and sodium chloride (placebo) at doses of 60 mg in 1 mL, 5 mg, and 0.9%, respectively, administered over 15 min. The primary outcome of interest is changes in femoral neck BMD for the year after ICU discharge. The estimated trial completion date is May 2025. Substantial future research is warranted to further explore the effectiveness of denosumab on bone health within the field of critical care.

### 6.4. Anabolic Agents

#### Teriparatide

Teriparatide, also known as recombinant human parathyroid hormone (PTH), stimulates bone formation and possesses potent anabolic properties [128]. In 2001, Neer and colleagues conducted an RCT that demonstrated the efficacy of PTH in the treatment of postmenopausal osteoporosis. PTH administration was shown to increase BMD in the spine, femur, and whole body while reducing the risk of both vertebral and non-vertebral fractures [129]. A systematic review and meta-analysis including 22 RCTs demonstrated the efficacy of teriparatide in reducing the incidence of hip fractures by 56% among patients with osteoporosis [130]. Due to associated adverse events such as hypercalcemia and osteosarcoma, contraindications for teriparatide include known or suspected Paget’s disease, previous bone radiotherapy, a medical history of hypercalcemia, renal disorders, and primary hyperparathyroidism [131]. Although no studies have focused solely on the effectiveness of teriparatide for treating ICU patients, the current evidence suggests that anti-resorptive therapy may be more appropriate than bone formation agents for mitigating fracture risk following acute critical illness [44]. Table 1 summarizes the aforementioned pharmacological modalities for treating or mitigating the effects of bone loss in critically ill patients.

## 7. Novel Approaches and Future Considerations in the Treatment of Osteoporosis

### 7.1. Combination Regimens

Research regarding the therapeutic efficacy of combination regimens for the treatment of osteoporosis has yielded promising results. A systematic review and meta-analysis by Lou et al. demonstrated a 36% advantage (compared with monotherapy regimens) brought about by combination therapy with PTH analogs and anti-resorptive agents in reducing fracture risk among patients with osteoporosis. Moreover, combination regimens showed considerable advantages in increasing lumbar and total hip BMD without serious adverse events [132]. The combination of teriparatide with denosumab was evaluated in the Denosumab and Teriparatide Administration (DATA) trial [133]. Relative to the monotherapy group, BMD increases in the hip and spine were observed in the first year of therapy; this rise in BMD was maintained but not increased during the second year of combination therapy. Although this study showed the potential benefits of combination therapy, the sample size was too small to determine a fracture risk reduction relative to conventional monotherapy [134]. Therefore, cumulatively, these findings support sequential administration for the treatment of osteoporosis and continuous treatment with bisphosphonates or denosumab for patients at high risk of fractures [134].

### 7.2. Novel Pharmaceutical Approaches

#### 7.2.1. Romosozumab

Romosozumab has both anabolic and anti-resorptive effects due to its inhibition of sclerostin [135]. Sclerostin, a protein emanating from mature osteocytes, exerts its inhibitory influence by blocking the activation of the Wnt/β-catenin osteogenic pathway within osteoblasts, resulting in a reduction in the process of bone formation [136]. A meta-analysis including nine RCTs investigated the efficacy of romosozumab in comparison with a placebo (alendronate) and teriparatide in the treatment of osteoporosis in postmenopausal patients [137]. Romosozumab demonstrated substantial efficacy in the treatment of osteoporosis and fractures. However, romosozumab was also noted to elevate the risk of hypersensitivity and adverse reactions at the injection site. The Active Controlled Fracture Study in Postmenopausal Women with Osteoporosis at High Risk (ARCH) trial demonstrated a slight increase in cardiovascular adverse events in patients receiving romosozumab. Although more supporting data are required, this treatment modality is not recommended for patients with a high risk of myocardial infarction or other cardiovascular events [138]. Romosozumab, approved by the U.S. Food and Drug Administration in 2019, has a novel mechanism of action. While the dual mechanism attributes have potential therapeutic benefits, future investigations are needed to further the understanding and clarify the therapeutic potential of this agent.

#### 7.2.2. Donepezil

Commonly prescribed to patients with dementia and administered to patients with Alzheimer’s disease, donepezil—with its acetylcholinesterase-inhibiting properties—has shown promise in the treatment of osteoporosis [139]. Acetylcholine plays a functional role in bone formation and in the regulation of cell matrix interactions in bone tissue, suggesting acetylcholinesterase as a potential molecular target for osteoporosis treatment [140]. A case–control study conducted in Spain evaluated the therapeutic benefits of donepezil and the risk of hip fracture among patients with Alzheimer’s disease. Donepezil was associated with beneficial effects on bone turnover, and the study concluded that donepezil administration was associated with reduced hip fracture risk among patients with Alzheimer’s disease [139].

#### 7.2.3. Regenerative Medicine and Stem Cell Therapy

Bone formation, including fracture repair and remodeling, requires the presence of mesenchymal stem cells (MSCs). These pluripotent cells replicate continuously, and they differentiate into various lineages, such as bone, cartilage, and ligamentous tissue [141]. The associated intricate molecular pathways involve cellular transitions and various molecular factors in the cellular progression and transformation. Regenerative medicine, which includes stem cell-based therapies, is gaining recognition in the treatment of various medical conditions, including osteoporosis, as these modalities can enable personalized and tailored regenerative treatment options. Stem cells are considered an ideal candidate for cell therapy in the treatment of osteoporosis owing to their plasticity and self-renewal properties. Several types of stem cells have been considered as treatment options for osteoporosis, including embryonic stem cells, induced pluripotent stem cells, and MSCs [142]. Compared with other stem cells, MSCs are more accessible and are much easier to harvest; this is in addition to their superior immunosuppressive properties and a relative lack of ethical concerns, which make these cells a viable option for bone regeneration treatment [143,144]. The bone repair molecular cascade is regulated by various signals from cytokines and growth factors from osteoprogenitor cells and the bony environment [145]. MSCs secrete biomolecules, such as transforming growth factor beta (TGF-β), insulin-like growth factor 1 (IGF-1), vascular endothelial growth factor (VEGF), hepatocyte growth factor (HGF), angiogenin, and interleukin 6, which enhance bone regeneration [146,147]. Exosomes derived by MSCs have also demonstrated molecular effects in osteogenesis, osteoclastogenesis, and in the prevention of bone loss in vitro and in vivo [148,149].

MSC transplantation represents a viable approach, and its impact on bone development has been previously documented [150]. MSCs possess the ability to directly address the affected regions and also transform into osteoblasts, which is their inherent role in the formation of bone. Particularly, MSCs derived from bone marrow exhibit a high potential for osteogenic differentiation and are regarded as a dependable and efficient resource for treating osteoporosis through MSC therapies [151,152]. The transplantation of MSCs has been carried out in both animal models and humans with osteoporosis, demonstrating the potential to enhance the local environment, stimulate bone regeneration, and exert paracrine effects. Consequently, the bioactive molecules released by MSCs, including their secretomes and exosomes, may play key roles in regulating osteoporosis, complementing the therapeutic benefits of the cell-based approach [153].

Osteoblast differentiation is regulated by two transcription factors, the runt-related transcription factor 2 (RUNX2) and osterix (Osx). The activation or inhibition of these transcription factors plays a significant role during osteogenic differentiation in MSCs. In addition, micro RNA regulators are essential in the differentiation of stem cells, as these micromolecules suppress bone cell formation while promoting the formation of adipocytes [154]. With the therapeutic limitations of conventional osteoporosis treatment, the necessity to continue adapting this type of therapy is warranted, and further understanding of controlling cell migration of implanted MSCs is mandatory. The stem cells lack the ability to recognize the treatment-required bone surfaces, which have the potential to differentiate and mature to an unspecific, non-osteogenic graft, which will be problematic to maintain and utilize in the clinical setting [155].

## 8. Limitations in the Treatment of Osteoporosis in the Intensive Care Setting

Due to their complex medical history, ICU survivors are a specific population with individualized needs and unique limitations. However, after hospital discharge, these patients may be neglected in the healthcare system with difficulties in accessing clinical care that addresses their medical situation. 

### 8.1. Managing Therapeutic Compliance in ICU Patients 

Despite the negative impact of osteoporosis, not all high-risk fracture patients have access to treatment modalities and facilities, which include pharmacological treatment of osteoporosis [118]. High-risk patients can be lost or unidentified in the healthcare system, which results in a disparity in osteoporosis management [156]. In the clinical setting, physicians experience poor compliance with prescribed medication in osteoporosis patients. Due to the asymptomatic nature and slow disease progression of osteoporosis, maintenance pharmacotherapy provides little or negligible therapeutic relief or benefit, which results in a low compliance rate in this disease population [118]. Other factors for the low adherence to pharmacotherapy are the economic burden, the cost of treatment regimens, and adverse events due to drug intake. Long-term treatment with bisphosphonates is generally considered safe and effective. However, due to adverse events such as gastrointestinal irritation, patients discontinue bisphosphonate treatment. In the most severe cases, reactions in the upper gastrointestinal tract may also include esophageal erosion or esophageal ulcers [118]. 

### 8.2. Multidisciplinary Approach in the Management of Post-ICU Survivors

Although current and recommended treatment guidelines for adult survivors of sepsis improve survival outcomes, only a select number of patients undergo these treatment practices [157]. Therefore, there is a practical need for a dedicated, multidisciplinary treatment service for the management of ICU survivors. A recent systematic review concluded that this approach has shown potential benefits [158]. However, results from the Pragmatic Randomized Controlled Trial of Intensive Care (PRaCTICaL) study and the Increased Hospital-Based Physical Rehabilitation and Information Provision after Intensive Care Unit Discharge (RECOVER) randomized controlled trial showed no therapeutic benefit or positive outcomes [159,160]. 

Since there are no practical definitions for post-ICU management, treatment protocols show numerous variations in medical personnel, patient inclusion, and follow-up duration. There are no treatment standards or guidelines for the selection of ICU survivors that should be included in follow-up programs. Medical personnel, such as intensive care specialists and physicians with experience and interest in this clinical field, should be considered the most qualified to understand the prognostic aspects of critically ill patients and play a vital role in the design and supervision of the aforementioned services.

### 8.3. Novel Management Strategies for Post-ICU Management

The utilization of telemedicine is another viable approach for managing post-ICU patients. Social media platforms such as text messaging or applications have shown potential as a method of communication and information sharing between healthcare providers and patients. Mobile phone applications with training programs have been shown to be effective in mitigating psychological distress in ICU survivors and family members [161]. Telemedicine gives specialty practitioners an advantage because they can observe the patients in their home environment and allow the physician to provide tailored support to the patient. Moreover, this media is increasingly used for peer support within support groups of ICU survivors [161].

In addition to addressing the diagnosis and treatment of bone loss of the critically ill, it is crucial for post-ICU care to focus on preventing readmissions [162]. Early identification of survivors who are at high risk of recurrence is essential. Regular reassessment of medications is necessary, as vital parameters may fluctuate in the weeks following hospital discharge, potentially requiring adjustments to drug types or doses. Moreover, educating vulnerable patients about the risk of reinfection is important, and assessing deglutition function is also recommended.

The Fracture Liaison Service (FLS) model seems to effectively address several historical deficiencies in the traditional approach to managing fragility fractures [163]. It has demonstrated success in enhancing diagnosis, optimizing long-term treatment, and reducing morbidity in individuals with such fractures. This model also eliminates uncertainty regarding the responsible medical specialty and facilitates streamlined communication among various specialties, minimizing the risk of patients becoming lost in the complexities of the current healthcare system. With the aging population, the imperative to manage and prevent life-altering fractures is growing. Recognizing that fractures are often the first sign of osteoporosis, collaboration between orthopedic surgeons and primary care physicians is crucial to initiate and guide the initial management of these patients. 

## 9. Conclusions

Significant strides have been made in comprehending bone loss associated with critical illness over the past decade. Consistent evidence now supports the presence of aberrant bone metabolism in critical illness, characterized by an initial surge in bone resorption and concurrent suppression of bone formation, a pattern that endures for up to a month. Subsequent alterations in bone resorption and heightened formation become apparent in the following year. Notably, heightened bone turnover during critical illness has negative outcomes, including diminished BMD and an elevated risk of fractures over subsequent years. Early indications suggest that interventions targeting fractures may hold promise in mitigating bone loss and decreasing mortality after recovery from critical illness.

However, further research is required to elucidate the breadth of these effects across the spectrum of disease. Research to advance our understanding of the collective influence of critical illness on the spectrum of bone metabolism is essential. The discernment of major determinants of bone health in critical illness requires expansive participant cohorts, ideally with meticulously curated pre-critical illness data—a task of considerable magnitude in critical care research. Other important areas of investigation are the classification of biological pathways implicated in bone loss, the interplay of cytokine pathways with bone turnover, and molecular cascades involving bone metabolism (a domain only just beginning to be elucidated). Delving deeper into these mechanisms may provide insight into novel treatment modalities that impact both bone health and inflammatory outcomes.

The discernment of osteopenia and osteoporosis in the intensive care setting, as opposed to the general aging issues of the population, marks a significant stride. Critically ill patients require a specialized approach to address osteoporosis, distinct from the general elderly population. The unique challenges of bone loss in critical illness, influenced by multifactorial factors such as the inflammatory response, immobilization, and the use of certain medications, emphasize the need for tailored interventions. Recognizing and managing osteoporosis in this context demand a nuanced understanding of the complex interventions during critical illness, guiding the development of effective preventive and treatment strategies.

## Figures and Tables

**Table 1:** Pharmacologic interventions for osteoporosis in critically ill patients.

Class	Drugs	Dose Adjustment	Regimen	Side Effect
Nutrient				
Vitamin D	Vitamin D2/D3	No	600–2000 IU PO daily	Hypercalcemia
Anti-resorptive agents				
Bisphosphonates	Alendronate	Dose adjustment in kidney impairment	10 mg PO daily or 70 mg PO weekly	Hypocalcemia GI mucosal irritation Osteonecrosis of the Jaw Atypical Femoral Fractures
	Ibandronate	Contraindicated in kidney impairment	150 mg PO monthly or 3 mg IV every 3 months	
	Risendronate		5 mg PO daily, 35 mg PO weekly, or 150 mg PO monthly	
	Zoledronic acid		Monthly/every 3 months Daily/weekly/monthly One time per year	Atrial fibrillation Hypocalcemia GI mucosal irritation
RANKL inhibitor	Denosumab	No	60 mg SC every 6 months	Hypocalcemia Infection
Anabolic agents				
PTH analog	Teriparatide	No	20 µg SC daily	Hypercalcemia Osteosarcoma
Scclerostin inhibitor	Romosozumab	No	210 mg monthly for 12 months	Myocardial infarction Stroke Cardiovascular death

RANKL, receptor activator of nuclear factor-kappa B ligand; PO, per os; SC, subcutaneous; GI, gastrointestinal; PTH, parathyroid hormone.

## Data Availability

Data sharing is not applicable.
